# Gemfibrozil Induces Anemia, Leukopenia and Reduces Hematopoietic Stem Cells via PPAR-α in Mice

**DOI:** 10.3390/ijms21145050

**Published:** 2020-07-17

**Authors:** Gabriel Rufino Estrela, Adriano Cleis Arruda, Heron Fernandes Vieira Torquato, Leandro Ceotto Freitas-Lima, Mauro Sérgio Perilhão, Frederick Wasinski, Alexandre Budu, Ricardo Ambrósio Fock, Edgar Julian Paredes-Gamero, Ronaldo Carvalho Araujo

**Affiliations:** 1 Department of Clinical and Experimental Oncology, Discipline of Hematology and Hematotherapy, Federal University of São Paulo, São Paulo 04037002, Brazil; 2 Department of Medicine, Discipline of Nephrology, Federal University of São Paulo, São Paulo 04039032, Brazil; arruda_adriano@hotmail.com (A.C.A.); maurospersonal3@gmail.com (M.S.P.); 3 Department of Biophysics, Federal University of São Paulo, São Paulo 04039032, Brazil; lcf.lima@gmail.com (L.C.F.-L.); alexandre.budu@unifesp.br (A.B.); 4 Department of Biochemistry, Federal University of São Paulo, São Paulo 04044020, Brazil; heron.fvt@gmail.com (H.F.V.T.); edgar.gamero@ufms.br (E.J.P.-G.); 5 Faculty of Pharmacy, University Center Braz Cubas, Mogi das Cruzes 08773380, Brazil; 6 Department of Physiology, Institute of Biomedical Sciences, University of São Paulo, São Paulo 05508000, Brazil; frednefro@gmail.com; 7 Department of Clinical and Toxicological Analysis, School of Pharmaceutical Sciences, University of São Paulo, São Paulo 05508000, Brazil; hemato@usp.br; 8 Faculty of Pharmaceutical, Sciences, Food and Nutrition, Federal University of Mato Grosso do Sul, Campo Grande, Mato Grosso do Sul 79070900, Brazil

**Keywords:** gemfibrozil, anemia, leukopenia, PPAR-alpha, hematopoietic stem cells, erythropoiesis

## Abstract

Hypercholesterolemia, also called high cholesterol, is a form of hyperlipidemia, which may be a consequence of diet, obesity or diabetes. In addition, increased levels of low-density lipoprotein (LDL) and reduced levels of high-density lipoprotein (HDL) cholesterol are associated with a higher risk of atherosclerosis and coronary heart disease. Thus, controlling cholesterol levels is commonly necessary, and fibrates have been used as lipid-lowering drugs. Gemfibrozil is a fibrate that acts via peroxisome proliferator-activated receptor alpha to promote changes in lipid metabolism and decrease serum triglyceride levels. However, anemia and leukopenia are known side effects of gemfibrozil. Considering that gemfibrozil may lead to anemia and that gemfibrozil acts via peroxisome proliferator-activated receptor alpha, we treated wild-type and peroxisome proliferator-activated receptor alpha-knockout mice with gemfibrozil for four consecutive days. Gemfibrozil treatment led to anemia seven days after the first administration of the drug; we found reduced levels of hemoglobin, as well as red blood cells, white blood cells and a reduced percentage of hematocrits. PPAR-alpha-knockout mice were capable of reversing all of those reduced parameters induced by gemfibrozil treatment. Erythropoietin levels were increased in the serum of gemfibrozil-treated animals, and we also observed an increased expression of *hypoxia-inducible factor-2 alpha* (*HIF-2α*) and *erythropoietin* in renal tissue, while PPAR-alpha knockout mice treated with gemfibrozil did not present increased levels of serum erythropoietin or tissue HIF-2α and erythropoietin mRNA levels in the kidneys. We analyzed bone marrow and found that gemfibrozil reduced erythrocytes and hematopoietic stem cells in wild-type mice but not in PPAR-alpha-knockout mice, while increased colony-forming units were observed only in wild-type mice treated with gemfibrozil. Here, we show for the first time that gemfibrozil treatment leads to anemia and leukopenia via peroxisome proliferator-activated receptor alpha in mice.

## 1. Introduction

One-third of ischemic heart disease is associated with high cholesterol, which is also related to increased risk of stroke and heart disease. Moreover, hypercholesterolemia is related to 2.6 million deaths, according to the World Health Organization. In 2008, the prevalence of increased cholesterol among adults was 39%. More than half of United States adults (55%, or 43 million) who could benefit from cholesterol medicine are currently using it [1].

Gemfibrozil is a fibrate used for the management of dyslipidemia. It is well-known to decrease low-density lipoprotein (LDL) and increase high-density lipoprotein (HDL) cholesterol, and it can also be used to reduce the development of coronary heart disease [2,3,4,5,6,7,8]. Gemfibrozil activates peroxisome proliferator-activated receptor alpha (PPAR-α), which leads to changes in lipid metabolism, decreasing serum triglyceride levels by modulating the lipoprotein lipase in adipose and muscle tissues [9]. Some side effects are associated with the use of fibrates, and anemia is one of them [10,11].

Anemia is characterized by a reduced ability of the blood to carry oxygen, and reduced levels of hemoglobin are observed in the peripheral blood, which usually also reflects the reduced number of red blood cells (RBCs). Moreover, anemia is the most common blood disorder, affecting about one-third of the global population [12]. It can be caused by blood loss, decreased red-blood-cell production and increased red-blood-cell breakdown [13]. It increases medical costs and lowers a person’s productivity through decreased ability to work [14].

Leukopenia is the term for a low level of white blood cells, or leukocytes, which are responsible for systemic defense against infections and diseases. Leukopenia can be caused by chronic conditions, infections and some drugs that slow bone-marrow function, leading to a reduced white-blood-cell count. The greatest danger of this condition is susceptibility to infections [15].

Considering that the prevalence of hyperlipidemia is high and that treatment with fibrates is frequent, we decided to establish a reliable model for studying gemfibrozil-induced anemia and check whether this effect was mediated by PPAR-α.

## 2. Results

### 2.1. PPAR-α Deletion Prevents Gemfibrozil-Induced Anemia

**Figure 1:**
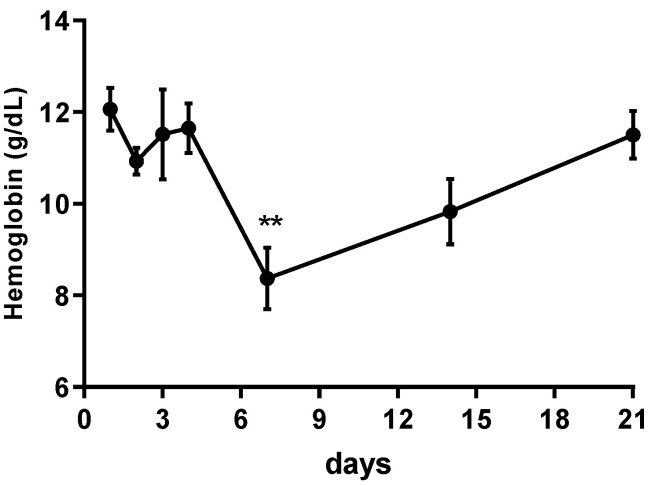
Hemoglobin levels during gemfibrozil treatment. The panel shows the hemoglobin levels at 7 different times over 21 days in WT mice. Data are presented as mean ± SEM; *n* = 6. One-way ANOVA) followed by Dunnett’s post hoc test. ** *p* < 0.01.

We treated wild-type (WT) mice for four consecutive days with gemfibrozil (150 mg/kg) by oral gavage and checked hemoglobin levels every day. Seven days after the first administration, we observed a huge decrease in hemoglobin levels (Figure 1). Seven days after the start of gemfibrozil treatment, WT mice developed anemia, with decreased levels of hemoglobin, decreased percentage volume of hematocrit, and decreased RBC and WBC counts (Table 1). PPAR-α-knockout mice treated with gemfibrozil avoided the decrease in hemoglobin levels, attenuated the lower percentage volume of hematocrit and blunted the lower RBC counts (Table 1).

### 2.2. PPAR-α Deletion Blunts Gemfibrozil-Induced Increase in Serum Erythropoietin

**Figure 2:**
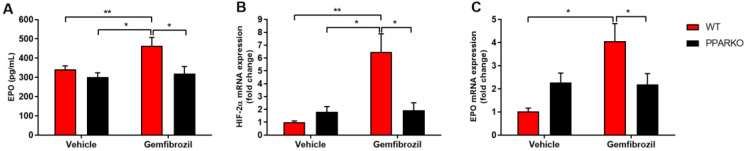
Gemfibrozil treatment increased renal erythropoiesis. Serum erythropoietin (EPO) levels (**A**) were increased with gemfibrozil treatment. Renal hypoxia-inducible factor-2 alpha (*HIF-2α*); (**B**) and *EPO*; (**C**) mRNA levels also increased after gemfibrozil treatment. Data are presented as mean ± SEM; *n* = 5–6 per group. Two-way ANOVA followed by Tukey’s post hoc test. * *p* < 0.05; ** *p* < 0.01.

Erythropoietin (EPO) is a glycoprotein that regulates the formation of erythrocytes and is mainly secreted by the kidneys in response to cellular hypoxia. Additionally, it stimulates red-blood-cell production in the bone marrow. Gemfibrozil treatment increased serum erythropoietin levels in WT mice, while PPAR-α deletion blunted this increase (Figure 2A).

### 2.3. PPAR-α-Knockout Mice Avoided the Increase in HIF-2α and Erythropoietin mRNA Levels Induced by Gemfibrozil in Renal Tissue

Hypoxia-inducible factor (HIF) is a transcription factor that responds under low oxygen availability [16]. HIF-2α is the main regulator of erythropoietin production [17]. Gemfibrozil-treated mice presented increased levels of *HIF-2α* and *EPO* mRNA in the renal tissue (Figure 2B,C), while PPAR-α deletion blunted this increase (Figure 2B,C).

### 2.4. PPAR-α Ablation Prevents Gemfibrozil-Induced Decreases in Erythroid and Hematopoietic Stem Cells in the Bone Marrow

**Figure 3:**
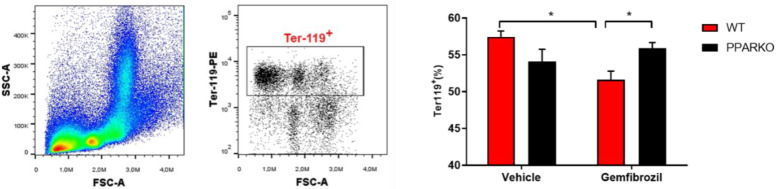
Gemfibrozil treatment decreased erythroid cells, and PPAR-alpha deletion blunted it. Ter-119+ is an erythroid marker. Gating strategy for Ter-119+ and quantification are presented. Data presented as mean ± SEM; *n* = 5–6 per group. Two-way ANOVA followed by Tukey’s post hoc test. * *p* < 0.05.

**Figure 4:**
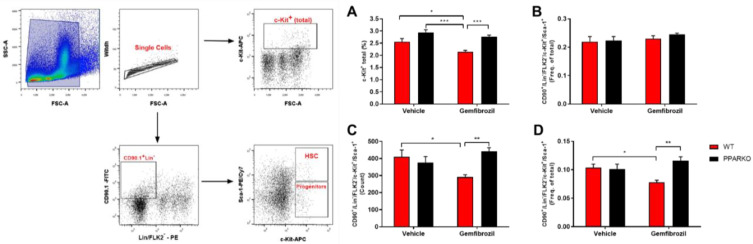
Gemfibrozil treatment reduced hematopoietic stem cells in the bone marrow, and PPAR-alpha deletion reversed it. Gating strategy for HSCs and progenitor cells is presented. Gemfibrozil reduced c-Kit^+^; (**A**) Frequency of progenitors showed no difference; (**B**) Gemfibrozil decreased HSC count; (**C**) and total frequency; (**D**) while PPAR-alpha deletion blunted these effects of gemfibrozil. Data presented as mean ± SEM; *n* = 5–6 per group. Two-way ANOVA followed by Tukey’s post hoc test. * *p* < 0.05; ** *p* < 0.01; *** *p* < 0.001.

Bone marrow is the primary hematopoiesis site; it is where all blood and immune cells are formed. TER-119 is an erythroid-specific marker expressed at all differentiation stages, from early proerythroblasts to mature erythrocytes [18]. Gemfibrozil decreased the percentage of TER-119 in the bone marrow, while PPAR-alpha-knockout mice maintained this percentage at a level similar to that of vehicle-treated WT mice, increasing the percentage compared to WT gemfibrozil (Figure 3). Hematopoietic stem cells (HSCs) were defined as Lin-FLK-2-Sca-1^+^c-Kit^+^Thy1.1^low^ [19]. Gemfibrozil treatment diminished HSC counts, while PPAR-alpha deletion was capable of reversing this reduction in the bone marrow (Figure 4A–D).

### 2.5. PPAR-α Deletion Blunted the Increased Levels of Colony Forming Units that Generate Myeloid Cells Induced by Gemfibrozil

**Figure 5:**
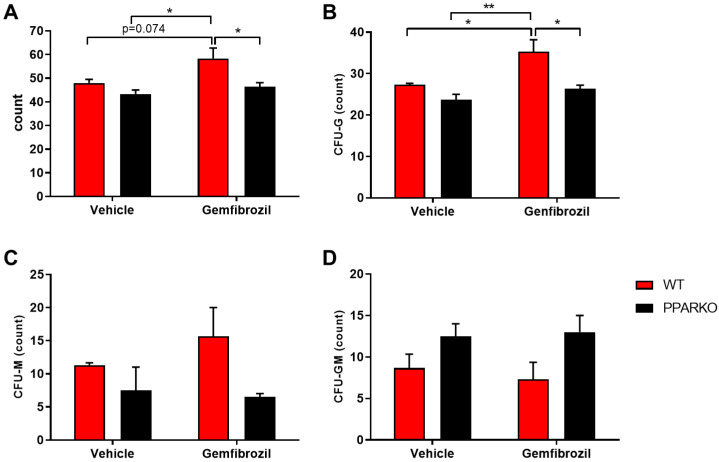
Gemfibrozil treatment increased total colony-forming units in the bone marrow. Gemfibrozil treatment tended to increase total CFU count; (**A**) CFU-granulocyte also increased after gemfibrozil treatment; (**B**) No statically significant differences were found in CFU-M (**C**) nor CFU-GM; (**D**) Moreover, PPAR-alpha deletion blunted these effects of gemfibrozil. Data are presented as mean ± SEM; *n* = 4–5 per group. Two-way ANOVA followed by Tukey’s post hoc test. * *p* < 0.05; ** *p* < 0.01.

It was found that gemfibrozil increased total colony-forming-unit (CFU) counts, while PPAR-α deletion kept CFU counts at the same levels as those observed in the vehicle group of WT mice (Figure 5A). We also found differences in granulocyte colony-forming units: gemfibrozil increased their counts and PPAR-α-knockout mice showed no changes (Figure 5B), maintaining the same levels as those of vehicle-treated WT mice. No differences were found in monocyte colony-forming units or colony-forming units of granulocyte-macrophage progenitor cells (Figure 5C,D).

## 3. Discussion

The prevalence of hyperlipidemia in the modern world is high, thus, the use of hypolipemiant drugs is increasing every day. However, the side effects must be better understood in order to find new tools to avoid them. Gemfibrozil is a fibrate, and it is used to activate PPAR-α [20,21]. Here, we developed a reliable model to study gemfibrozil-induced anemia in mice. We observed that gemfibrozil led to anemia seven days after the first administration and that hemoglobin levels diminished 14 days after the first dose and normalized 21 days later. In a study, after four weeks of 100 mg/kg bw/day of fenofibrate, rats developed slight anemia [10]. In our model, the development of anemia was clear, and we could see a reduction in all parameters analyzed by complete blood count. In order to better understand its mechanism, we analyzed renal tissue, where hypoxia-inducible factor has an important role when low oxygen levels are detected, and HIF-2α is crucial for erythropoietin induction [17].

HIF-2α-knockout mice presented anemia, which was not the result of a cell-autonomous defect in erythroid-precursor maturation but of inadequate renal EPO production; moreover, the location of renal HIF-2α-expressing interstitial cells coincides with that of renal EPO-producing cells [22,23,24]. Although hemoglobin, hematocrit and RBCs decreased, indicating an anemic condition in our mice, *HIF-2α* and *EPO* mRNA levels increased in the renal tissue, and increased serum EPO levels were observed with gemfibrozil treatment, which suggests that a compensatory mechanism was triggered to increase EPO production. This explains the normalization of hemoglobin levels on Day 21 after gemfibrozil treatment. Additionally, leukopenia, a common side effect of gemfibrozil treatment found in humans, was observed in gemfibrozil-treated mice [11]. Bone-marrow analyses corroborated blood analyses, in which we found decreased hematopoietic-stem-cell counts in addition to a decreased percentage of erythrocyte markers. Our results suggest that gemfibrozil may present some cytotoxicity. However, it has already been shown that fibrates can alter cell-cycle distributions, increasing G0/G1 phase and reducing G2/M phase, which shows that fibrates reduce cell proliferation with no or very low cytotoxic effects [25]. Further investigation of the mechanism that reduces the number of hematopoietic progenitor cells and the erythroid population through investigating cell arrest and cell-death mechanisms is necessary. There are no mechanisms described for gemfibrozil that alter hematopoiesis, but the inhibition of Stat-3, an important transcription factor in myelopoiesis, by gemfibrozil was observed in adipose and hepatic tissues [26].

The colony-forming-unit assay is widely used for analyses of hematopoietic and progenitor cells. It allows for the measurement of cell differentiation and proliferation. Both total CFU counts and CFU-granulocyte counts were increased in gemfibrozil-treated animals, showing that the bone marrow of gemfibrozil-treated mice increased the production of granulocyte colonies in order to potentially reverse leukopenia.

Here, we presented for the first time that gemfibrozil led to anemia in mice, decreasing erythrocytes and hematopoietic stem cells in the bone marrow and increasing signaling for RBC production in the renal tissue and WBC production in the bone marrow in order to compensate for these adverse effects. Moreover, we also showed that this effect of gemfibrozil was dependent on PPAR-alpha, since its ablation could reverse all these side effects. Additional studies are necessary to further clarify the mechanisms of gemfibrozil-induced anemia, but we showed that gemfibrozil effects were dependent on PPAR-α.

## 4. Methods

### 4.1. Animals

Wild type (WT, C57/BL6J) and PPAR-α knockout (PPARα KO, B6; 129S4-Pparatm1Gonz/J, Jackson laboratory) male mice weighing 23–27 g and aged 9–12 weeks, were used for these experiments. The animals were obtained from the Animal Care Facility of the Federal University of São Paulo (UNIFESP). All animals were housed in individual, standard cages and had free access to water and food. All procedures were previously reviewed and approved by the internal ethics committee of the Federal University of São Paulo (CEUA 6823010319).

### 4.2. Experimental Protocol

The mice were divided into 4 groups for each experiment: WT vehicle group, PPARKO vehicle group, WT gemfibrozil group and PPAR-α-KO gemfibrozil group. We used *n* = 5–6 for each experiment and condition.

### 4.3. Drug Treatment

Gemfibrozil was given by oral gavage (150 mg/kg) diluted in 0.5% methylcellulose [27,28,29,30]. The mice were treated for 4 days with gemfibrozil and euthanized 1 week after the beginning of treatment or followed for 21 days. Vehicle-group mice were given the same volume with 0.5% methylcellulose.

### 4.4. Blood Sampling and Tissue Collection

The mice were anesthetized with ketamine (91 mg/kg) and xylazine (9.1 mg/kg) intraperitoneally and blood was collected via cardiac puncture. Blood samples for serum collection were allowed to clot for 2 h at room temperature and then centrifuged for 20 min at 2000× *g*. The samples were then stored at –20 °C. Kidney tissue was collected, and the renal capsule was removed. Transversal cuts were performed, and the kidneys were immediately frozen in nitrogen and then stored at −80 °C.

### 4.5. Blood Count Test

Blood samples were collected with EDTA (Merck, Darmstadt, Germany), and blood counts were performed using ABX Micros ABC Vet equipment (Horiba ABX, Montpellier, France). Leukocyte differential and morphological analyses were performed on blood smears stained using the May–Grünwald–Giemsa (Merck, Darmstadt, Germany) technique.

### 4.6. Hemoglobin Measurement

The HemoCue portable system for hemoglobinometry can measure hemoglobin (Hb) concentration in less than one minute. Briefly, this device measures Hb by spectrophotometry using a small-volume (tailcut) optical-measuring cuvette and short light path. This device allows for the measurement of Hb concentration in blood samples several times and with low sample volumes.

### 4.7. Real-Time PCR

Kidney samples were frozen at –80 °C immediately after collection. Total RNA was isolated using TRIzol Reagent (Invitrogen, Carlsbad, CA, USA). The RNA integrity was assessed by electrophoresis on an agarose gel. cDNA was synthesized using the “High Capacity cDNA Reverse Transcription Kit” (Applied Biosystems). Standard curves were plotted to determine the amplification efficiency for each primer pair. Real-time PCR was performed with the SYBR Green system (Thermo Scientific, Waltham, MA, USA) using specific primers for *β-actin, 18S, EPO, HIF-2a*; the primers were designed using Primer3 web, and their specificity was confirmed using NCBI primer-BLAST; their sequences were *β-actin* forward 5′ CTGGCCTCACTGTCCACCTT 3′, reverse 5′ CGGACTCATCGTACTCCTGCTT 3′; *18s* forward 5′ CGCCGCTAGAGGTGAAATTC 3′, reverse 5′ TCTTGGCAAATGCTTTCGC 3′; *EPO* forward 5′ TCCACTCCGAACACTCACA 3′, reverse 5′ CCTCTCCCGTGTACAGCTT 3′; *HIF2a* forward 5′ CTGGACAAAGCCTCCATCAT 3′, reverse 5′ TTGCTGATGTTTTCCGACAG 3′. The cycling conditions were as follows: 10 min at 95 °C, followed by 45 cycles of 30 s at 95 °C, 30 s at 60 °C, and 30 s at 72 °C. Target mRNA expression was normalized to β-actin and 18s, and expressed as a relative value using the comparative threshold cycle (Ct) method (2−ΔΔCt). The expression levels of the genes of interest were normalized to the control group and presented as fold change.

### 4.8. Serum Erythropoietin Assay

Serum samples were frozen and stored at –20 °C immediately after collection. Serum EPO levels (KA1998) were quantified using the ELISA mouse kit specific to this analyte (Novus Biologicals, Littleton, CO, USA), according to the manufacturer’s instructions.

### 4.9. Colony-Forming-Unit Assay

Bone-marrow cells were collected from the mice’s femurs and suspended in Iscove’s Modified Dulbecco’s Medium (Invitrogen, Waltham, MA, USA). Twenty thousand cells were mixed into 1 mL of MethoCult GF M3434 (StemCell Technologies, Vancouver, BC, Canada) and supplemented according to the manufacturer’s instructions. The CFU assay was performed by placing cells in a 35-mm Petri dish. Cells were cultured in a humidity chamber at 37 °C with 5% CO_2_ for 14 days. At the end of the incubation period, colonies of more than 50 cells were counted using an inverted microscope at 20× magnification.

### 4.10. Immunolabeling

Hematopoietic cell populations were labeled with antibodies as previously described [31,32,33]. The following monoclonal antibodies were used to define the different hematopoietic cell populations: Ter-119 (Ly-76) and Mac-1 (M1/70). The cells were incubated with the antibodies for 30 min and then washed. In order to identify HSC, whole bone marrow cells (1 × 10^6^/sample) were labeled with a mature lineage (Lin) antibody cocktail (Gr1-PE, Mac-1-PE, CD3e-PE, Ter-119-PE and B220-PE), CD90.1 (Thy1.1.)-FITC, FLK-2-PE, Sca-1-PE/Cy7 and c-Kit-APC for 30 min. The cells were then washed. Gr-1^+^ cells were defined as myeloid cells, and Ter-119^+^ (Gr-1-) cells were defined as erythroid cells. HSCs were defined as Lin^-^FLK-2^-^Sca-1^+^c-Kit^+^Thy1.1^low^ [19]. All antibodies were purchased from Becton Dickinson (Franklin Lakes, NJ, USA). All cytometry analyses in this study were performed with an Accuri C6 (Becton Dickinson, Franklin Lakes, NJ, USA) flow cytometer. Total bone marrow cells count and total labelled cells count are found in supplementary materials (Figure S1).

### 4.11. Statistical Analysis

All data are presented as mean ± SEM. Intergroup-difference significance was assessed by two-way analysis of variance (ANOVA) with Tukey’s correction for multiple comparisons. The value for statistical significance was established at *p* < 0.05. All statistical analyses were performed using GraphPad Prism 8 (GraphPad, La Jolla, CA, USA).

## Figures and Tables

**Table 1:** PPAR-alpha deletion reverses decreasement of blood count parameters.

	WT	PPARKO	WT	PPARKO
Parameters	Vehicle	Vehicle	Gemfibrozil	Gemfibrozil
Hemoglobin (g/dL)	12.55 ± 0.36	12.37 ± 0.91	09.52 ± 0.89 ^ac^	12.64 ± 0.43
Hematocrit (%)	33.18 ± 2.05	32.37 ± 1.41	23.30 ± 0.60 ^ac^	28.00 ± 1.00
RBC (x10^6^/mm^3^)	06.39 ± 0.42	06.38 ± 0.29	04.46 ± 0.11 ^abc^	05.49 ± 0.19
WBC (/mm^3^)	1885 ± 212	1680 ± 213	1133 ± 92.3 ^a^	1365 ± 88.0
PLT (/mm^3^)	404.0 ± 15.6	405.3 ± 23.3	351.0 ± 08.9	395.8 ± 15.9

Data are presented as mean ± SEM ^a^ vs. WT vehicle; ^b^ vs. PPARKO vehicle; ^c^ vs. PPARKO gemfibrozil; *p* < 0.05.

## Supplementary Materials

The following is available online at https://www.mdpi.com/1422-0067/21/14/5050/s1. Figure S1: Total bone marrow cell count and total flow cytometry marker count.

## Abbreviations

CFU	colony-forming unit
EPO	erythropoietin
HDL	high-density lipoprotein
HIF-2α	hypoxia-inducible factor 2 alpha
HSC	hematopoietic stem cell
LDL	low-density lipoprotein
PPAR-α	peroxisome proliferator-activated receptor alpha
RBC	red blood cells
WBC	white blood cells

## References

[1] Mercado C., DeSimone A.K., Odom E., Gillespie C., Ayala C., Loustalot F. (2015). Prevalence of cholesterol treatment eligibility and medication use among adults—United States, 2005–2012. MMWR Morb. Mortal. Wkly Rep..

[2] Jakob T., Nordmann A.J., Schandelmaier S., Ferreira-González I., Briel M. (2016). Fibrates for primary prevention of cardiovascular disease events. Cochrane Database Syst. Rev..

[3] Hankey G.J. (2002). Role of lipid-modifying therapy in the prevention of initial and recurrent stroke. Curr. Opin. Lipidol..

[4] Bloomfield R.H., Davenport J., Babikian V., Brass L.M., Collins D., Wexler L., Wagner S., Papademetriou V., Rutan G., Robins S.J. (2001). Reduction in stroke with gemfibrozil in men with coronary heart disease and low HDL cholesterol: The Veterans Affairs HDL Intervention Trial (VA-HIT). Circulation.

[5] Rubins H.B., Robins S.J., Collins D., Fye C.L., Anderson J.W., Elam M.B., Faas F.H., Linares E., Schaefer E.J., Schectman G. (1999). Gemfibrozil for the secondary prevention of coronary heart disease in men with low levels of high-density lipoprotein cholesterol. Veterans affairs high-density lipoprotein cholesterol intervention trial study group. N. Engl. J. Med..

[6] Athyros V.G., Papageorgiou A.A., Avramidis M.J., Kontopoulos A.G. (1995). Long-term effect of gemfibrozil on coronary heart disease risk profile of patients with primary combined hyperlipidaemia. Coron. Artery Dis..

[7] Bell H., Dittmeier G., Martinez L. (1988). Gemfibrozil therapy in patients with coronary heart disease. Mo. Med..

[8] Vega G.L., Grundy S.M. (1985). Gemfibrozil therapy in primary hypertriglyceridemia associated with coronary heart disease. Effects on metabolism of low-density lipoproteins. JAMA.

[9] Kersten S. (2008). Peroxisome proliferator activated receptors and lipoprotein metabolism. PPAR Res..

[10] Strauss V., Mellert W., Wiemer J., Leibold E., Kamp H., Walk T., Looser R., Prokoudine A., Fabian E., Krennrich G. (2012). Increased toxicity when fibrates and statins are administered in combination—A metabolomics approach with rats. Toxicol. Lett..

[11] Kim K., Kleinman H.K., Lee H.J., Pahan K. (2017). Safety and potential efficacy of gemfibrozil as a supportive treatment for children with late infantile neuronal ceroid lipofuscinosis and other lipid storage disorders. Orphanet J. Rare Dis..

[12] Chaparro C.M., Suchdev P.S. (2019). Anemia epidemiology, pathophysiology, and etiology in low- and middle-income countries. Ann. N. Y. Acad Sci..

[13] Janz T.G., Johnson R.L., Rubenstein S.D. (2013). Anemia in the emergency department: Evaluation and treatment. Emerg. Med. Pract..

[14] Smith R.E. (2010). The clinical and economic burden of anemia. Am. J. Manag. Care.

[15] Ing V.W. (1984). The etiology and management of leukopenia. Can. Fam. Physician.

[16] Smith T.G., Robbins P.A., Ratcliffe P.J. (2008). The human side of hypoxia-inducible factor. Br. J. Haematol..

[17] Haase V.H. (2013). Regulation of erythropoiesis by hypoxia-inducible factors. Blood Rev..

[18] Barminko J., Reinholt B., Baron M.H. (2016). Development and differentiation of the erythroid lineage in mammals. Dev. Comp. Immunol..

[19] Barbosa C.M., Leon C.M., Nogueira-Pedro A., Wasinsk F., Araújo R.C., Miranda A., Ferreira A.T., Paredes-Gamero E.J. (2011). Differentiation of hematopoietic stem cell and myeloid populations by ATP is modulated by cytokines. Cell Death Dis..

[20] Fruchart J.C., Duriez P., Staels B. (1999). Molecular mechanism of action of the fibrates. J. Soc. Biol..

[21] Schoonjans K., Staels B., Auwerx J. (1996). Role of the peroxisome proliferator-activated receptor (PPAR) in mediating the effects of fibrates and fatty acids on gene expression. J. Lipid Res..

[22] Paliege A., Rosenberger C., Bondke A., Sciesielski L., Shina A., Heyman S.N., Flippin L.A., Arend M., Klaus S.J., Bachmann S. (2010). Hypoxia-inducible factor-2alpha-expressing interstitial fibroblasts are the only renal cells that express erythropoietin under hypoxia-inducible factor stabilization. Kidney Int..

[23] Scortegagna M., Ding K., Zhang Q., Oktay Y., Bennett M.J., Bennett M., Shelton J.M., Richardson J.A., Moe O., Garcia J.A. (2005). HIF-2alpha regulates murine hematopoietic development in an erythropoietin-dependent manner. Blood.

[24] Rosenberger C., Mandriota S., Jürgensen J.S., Wiesener M.S., Hörstrup J.H., Frei U., Ratcliffe P.J., Maxwell P.H., Bachmann S., Eckardt K.-U. (2002). Expression of hypoxia-inducible factor-1alpha and -2alpha in hypoxic and ischemic rat kidneys. J. Am. Soc. Nephrol..

[25] Scatena R., Nocca G., Sole P.D., Rumi C., Puggioni P., Remiddi F., Bottoni P., Ficarra S., Giardina B. (1999). Bezafibrate as differentiating factor of human myeloid leukemia cells. Cell Death Differ..

[26] Hua H., Yang J., Lin H., Xi Y., Dai M., Xu G., Wang F., Liu L., Zhao T., Huang J. (2018). PPARα-independent action against metabolic syndrome development by fibrates is mediated by inhibition of STAT3 signalling. J. Pharm. Pharmacol..

[27] Blednov Y.A., Black M., Benavidez J.M., Stamatakis E.E., Harris R.A. (2016). PPAR agonists: II. fenofibrate and tesaglitazar alter behaviors related to voluntary alcohol consumption. Alcohol. Clin. Exp. Res..

[28] Calkin A.C., Giunti S., Jandeleit-Dahm K.A., Allen T.J., Cooper M.E., Thomas M.C. (2006). PPAR-alpha and -gamma agonists attenuate diabetic kidney disease in the apolipoprotein E knockout mouse. Nephrol. Dial. Transplant..

[29] Benke K., Mátyás C., Sayour A.A., Oláh A., Németh B.T., Ruppert M., Szabó G., Kökény G., Horváth E.M., Hartyánszky I. (2017). Pharmacological preconditioning with gemfibrozil preserves cardiac function after heart transplantation. Sci. Rep..

[30] Nesfield S.R., Clarke C.J., Hoivik D.J., Miller R.T., Allen J.S., Selinger K., Santostefano M.J. (2005). Evaluation of the carcinogenic potential of clofibrate in the rasH2 mouse. Int. J. Toxicol..

[31] Nogueira-Pedro A., Dias C.C., Regina H., Segreto C., Addios P.C., Lungato L., D’Almeida V., Barros C.C., Higa E.M., Buri M.V. (2014). Nitric oxide-induced murine hematopoietic stem cell fate involves multiple signaling proteins, gene expression, and redox modulation. Stem Cells.

[32] Nogueira-Pedro A., Barbosa C.M., Segreto H.R., Lungato L., D’Almeida V., Moraes A.A., Miranda A., Paredes-Gamero E.J., Ferreira A.T. (2011). α-Tocopherol induces hematopoietic stem/progenitor cell expansion and ERK1/2-mediated differentiation. J. Leukoc. Biol..

[33] Leon C.M., Barbosa C.M., Justo G.Z., Borelli P., Resende J.D., de Oliveira J.S., Ferreira A.T., Paredes-Gamero E.J. (2011). Requirement for PLCγ2 in IL-3 and GM-CSF-stimulated MEK/ERK phosphorylation in murine and human hematopoietic stem/progenitor cells. J. Cell Physiol..

