# Supplementary material for: Gemfibrozil Induces Anemia, Leukopenia and Reduces Hematopoietic Stem Cells via PPAR-α in Mice
Source: Int J Mol Sci. 2020 Jul 17;21(14):5050. doi: 10.3390/ijms21145050 (PMC7403977; doi:10.3390/ijms21145050)
Supplement: Supplementary file 1 [file ijms-21-05050-s001.pdf]

Supplementary figure S1

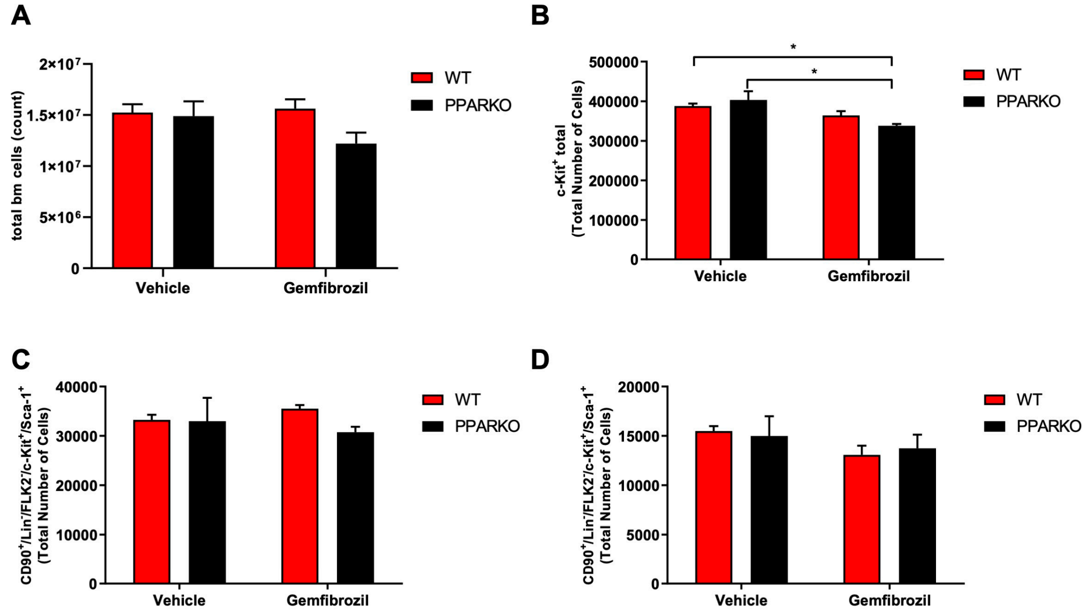

Supplementary Figure S1 | Total bone marrow cells count and total flow cytometry markers count. The panels show the total number of bone marrow cells (A), total number of c-Kit^{+} (B), total number of progenitors (CD90^{+}Lin^{-}c-Kit+Sca-1^{+}) (C) and total number of HSCs (D). Data presented as Mean ± SEM; n = 5-6 per group. Two-way ANOVA followed by Tukey's post-hoc test. * $p < 0.05$.
